# How Could Hospitalisations at the End of Life Have Been Avoided? A Qualitative Retrospective Study of the Perspectives of General Practitioners, Nurses and Family Carers

**DOI:** 10.1371/journal.pone.0118971

**Published:** 2015-03-10

**Authors:** Maria C. De Korte-Verhoef, H. Roeline W. Pasman, Bart P. M. Schweitzer, Anneke L. Francke, Bregje D. Onwuteaka-Philipsen, Luc Deliens

**Affiliations:** 1 Department of Public and Occupational Health & Expertise Center Palliative Care VUmc, EMGO Institute for Health and Care Research, VU University medical center (VUmc), Amsterdam, The Netherlands; 2 Department of General Practice, EMGO Institute for Health and Care Research, VU University medical center, Amsterdam, The Netherlands; 3 Netherlands Institute for Health Services Research, NIVEL, Utrecht, The Netherlands; 4 End-of-life Care Research Group, Ghent University & Vrije Universiteit Brussel, Brussels, Belgium

## Abstract

**Background:**

Although many patients prefer to stay and die at home at the end of life, many are hospitalised. Little is known about how to avoid hospitalisations for patients living at home.

**Aim:**

To describe how hospitalisation at the end of life can be avoided, from the perspective of the GPs, nurses and family carers.

**Method:**

A qualitative design with face-to-face interviews was used. Taking 30 cases of patients who died non-suddenly, 26 GPs, 15 nurses and 18 family carers were interviewed in depth. Of the 30 patients, 20 were hospitalised and 10 were not hospitalised in the last three months of life.

**Results:**

Five key themes that could help avoid hospitalisation at the end of life emerged from the interviews. The key themes were: 1) marking the approach of death, and shifting the mindset; 2) being able to provide acute treatment and care at home; 3) anticipatory discussions and interventions to deal with expected severe problems; 4) guiding and monitoring the patient and family in a holistic way through the illness trajectory; 5) continuity of treatment and care at home. If these five key themes are adopted in an interrelated way, this could help avoid hospitalisations, according to GPs, nurses and family carers.

**Conclusions:**

The five key themes described in this study can be seen as strategies that could help in avoiding hospitalisation at the end of life. It is recommended that for all patients residing at home, GPs and community nurses work together as a team from the moment that it is marked that death is approaching up to the end of life.

## Introduction

Hospitalisations at the end of life are a concern for many patients, because most of them prefer to stay at home and to be cared for there [1–3]. For instance, in the Netherlands and Belgium, more than half of the patients residing at home are hospitalised in the last three months of life and most of these hospitalised patients die in hospital [4,5]. A high proportion of hospitalisations at the end of life in the population is considered a problem because of the high healthcare expenditures [6] and because it is an indicator of poor quality in end-of-life care [7]. In general, the reasons most commonly given for the hospitalisation of patients at the end of life are somatic symptoms (such as dyspnoea, digestive, cardiovascular problems and pain), psychological problems (such as confusion or altered level of consciousness), or social problems (such as inability to cope at home) [8–10]. However, it has been suggested that these problems can often be managed at home [11].

For hospitalised patients with a short life expectancy, it has been estimated that 7% to 33% of hospitalisations could have been avoided, according to clinical experts’ assessments using hospital charts and professionals working in the hospital [8,11,12]. A focus group study among (clinical) professionals found that the main reasons for inappropriate hospitalisations were family carers being unable to cope at home, the ‘rescue culture’ of modern medicine, the costs of receiving community services and the availability of community services, and practice within the homes for elderly [13].

We acknowledge that hospitalisation at the end of life can be unavoidable or actually desirable for some patients, for instance when patients consider the hospital as a ‘safe haven’ or when an uncontrollable acute medical situation occurs at home [13,14]. However, because of the patients’ preferences and the quality of care, and also from an economic perspective, it is important to know more about the avoidability of hospitalisations from home. Only one focus group study has described the reasons for inappropriate hospitalisations [13]. To unravel the avoidability of hospitalisations from home, it is important to know what the situation was at home for the patients who were hospitalised and those who were not hospitalised, and how to avoid hospitalisation from the perspective of professionals providing care in the patient’s home and family carers. Therefore, this study aimed to describe how hospitalisation at the end of life can be avoided, from the perspective of GPs, community nurses and family carers in the Netherlands.

In the Netherlands, palliative care is generalistic in nature, which means that GPs and nurses are responsible for the palliative care of patients living at home. GPs have mostly known their patients for a long time and often collaborate with nurses [15]. Early in the disease process, nurses may provide the patient and their family with advice, instruction and information, which is often limited to five hours, on average, in the total illness process [16]. Nursing care to help with daily activities in the daytime can generally be provided up to a maximum of four hours a day. If the life expectancy is less than three months, night care can also be provided for eight hours a day. Additional specialised nursing care is available for technical care such as a syringe pump or infusion therapy. All professionals have the possibility to consult a specialised palliative care team when necessary.

## Methods

### Design

A qualitative descriptive study was conducted focusing on cases of deceased patients who resided at home, some of who were hospitalised and others who were not hospitalised at the end of life. Retrospective qualitative in-depth interview study was performed and analysed, using an inductive thematic approach [17], in the first half of 2012.

### Study population

This qualitative study is an addition to a broader Dutch national questionnaire study among GPs in the first half of 2011 [18]. The questionnaire included various items such as questions about the patient’s age and place of death, all diseases suffered by the patient, the cause of death, the main reason for hospitalisation and whether the patient was hospitalised in the last three months of life. In this questionnaire study, GPs were asked about the last deceased patient who died non-suddenly. The GP who received the questionnaire was asked to send a letter to the family carer inviting them to take part in the study. The questionnaire already included a question about which nurse (if any) was most closely involved in the care of the patient. This nurse was then also invited to take part in the study. For this qualitative study, we purposively selected 20 hospitalised patient cases based on the patients’ characteristics, such as age, gender and disease, and hospitalisation characteristics, such as the reason for hospitalisation. To contrast with the hospitalised patients we purposively selected 10 patient cases that were quite similar to the hospitalised patient cases but without hospitalisation in the last three months of life, in order to learn more about avoidability. This resulted in 59 in-depth interviews with 26 GPs, 18 family carers and 15 nurses.

### Data collection

Of the 59 in-depth interviews, 54 were held face to face at the preferred place of the respondent and five were held by telephone. The interviews lasted 60 minutes on average. The interviews started with a ‘grand tour’ question: “Tell me about the patient’s situation in the final three months of life for whom the GP filled in the questionnaire”. In the hospitalised patient cases, the interviewee was asked to talk about the circumstances surrounding the hospitalisation and about how hospitalisation could have been avoided or what would have helped the patient to stay at home. GPs and nurses were also asked about other recent cases where they thought that hospitalisation could have been avoided and, in contrast, about patients who were not hospitalised in the last three months of life. A short interview report with the main findings and a description of the respondent’s environment was written up after each interview. The in-depth interviews were conducted by a nurse scientist who has 15 years’ experience with qualitative research (MDK).

### Analyses

The interviews were recorded, transcribed verbatim and then read thoroughly by the first researcher (MDK) [19]. The coding scheme was inductively derived from the content of the interview transcripts. This was supported by the software program Atlas-ti. The codes were categorised into key themes that gave answers to the research question [17]. For each theme, we looked at the aspects that could be positively or negatively related to hospitalisations at the end of life. The perspectives of the GPs, the nurses and the family carers were all analysed. This qualitative analysis process was discussed step by step with one of the co-authors (HRP). Regular meetings were held with all researchers to discuss the findings, the coding scheme and the analyses.

### Ethics

A study protocol was approved by the Ethics Board of the VU University Medical Center Amsterdam. Before the start of each in-depth interview, the respondent was told that participation was voluntary, that the transcripts would be anonymous and that confidentiality was assured. After that, an informed consent form was signed by the respondent.

## Results

### Characteristics of respondents (Table 1)

The mean age of the 26 GPs interviewed was 50 (range 32–64). About a quarter of them were female. Of the 15 nurses interviewed, the mean age was 45 (range of 24–57), most were female and about half of them worked in a highly urbanised environment. The mean age of the family carers interviewed was 59 (range 44–82), most of them were female and three quarters of them were the patient’s partner. Of the 30 patient cases being discussed in the interviews, the mean age of the patients was 73 (range 49–97), one third of them were female, three quarters of them died of cancer.

**Table 1:** Characteristics of GPs, nurses, family carers and patient cases.

	GP	Nurse	Family carer	Patients’ cases
	(n = 26)	(n = 15)	(n = 18)	(n = 30)
Mean age (Range)	50 (32–64)	45 (24–57)	59 (44–82)	73 (49–97)
Gender, female	27%	93%	83%	30%
Urbanisation level				
Very high to high		34%	44%	40%
Relation to the patient				
Partner	x	x	72%	x
Son/daughter	x	x	22%	x
Friend	x	x	6%	x
Cause of death, cancer	x	x	x	73%
Hospitalised	x	x	x	67%

### Five key themes that could help avoid hospitalisation

The interviews with GPs, nurses and family carers revealed the complexity of the patients’ situation, which included the difficulty of identifying that death is approaching, the complex disease trajectory, the psychosocial circumstances and the communication between professionals. From these complex patient cases, five key themes emerged that could help avoid hospitalisation: 1) marking the approach of death, and shifting the mindset; 2) being able to provide acute treatment and care at home; 3) anticipatory discussions and interventions to deal with expected severe problems; 4) guiding and monitoring the patient and family in a holistic way through the illness trajectory; 5) continuity of treatment and care at home. These five themes are illustrated by exploring the positive and negative experiences of the participants in each of the five areas.

#### 1. Marking the approach of death, and shifting the mindset

From the interviews with GPs, nurses and family, it emerged that to avoid hospitalisation, physicians had to have concluded and clearly communicated that the patient had a short life expectancy. This led to a huge shift in the mindset of patients, family carers and professionals, which often needed some time to become a full awareness. This shift in the mindset gave a different view of treatment in the hospital, and then the benefits and burden of treatment in the hospital were weighed up in a different way. As long as there was a glimpse of hope of a cure or improvement, the burden of being in the hospital was taken for granted, but when improvement or cure could no longer be achieved then staying at home with the family became more important. When community nurses were involved, they felt their role was to reiterate and explain what had already been said about the incurability and short life expectancy to help the patient and their family develop a full awareness of the new reality, which was that no cure was possible and the end of life was approaching.

“Interviewer: Why is it necessary to say explicitly that someone is incurably ill?

Nurse: Well, of course it is necessary for the awareness and eventually for the acceptance, when that comes. Because of course people will otherwise… well ultimately they want to keep looking for something that will make them better. And that can get very difficult because that something is no longer an option.” (Case 6, Nurse, female, age category 45–50)

On the other hand, GPs often found it difficult to recognise and then mark that death was approaching. When it was not clear that death was imminent, then hospitalisations seemed less avoidable. Sometimes the physician had explained that death was approaching but the patient or the family still did not make a shift in their mindset to acknowledge that death was indeed approaching.

Interviewer: “What exactly did the specialist tell you and your husband in the hospital?”

Family carer: “That it was malignant, that they couldn’t cure it, only slow it down.

Interviewer: “What did you expect from the hospitalisation? Did your husband have certain expectations?”

Family carer: Well, really, you could really say he kept on hoping right up to the end that he might be cured. You know, just like my youngest daughter, she was also saying that you never know, do you?”

(Case 5, Family carer, female, age category 65–70)

This shift in the mindset could be difficult for some patients or their family to make. For them, it was important to know that everything was being done and therefore they went on with all possible treatments in the hospital; only when it finally became clear that no improvements could be achieved they were able to accept that death was approaching. One GP talked about a situation in which the patient and the family were not able to make a shift in the mindset to acknowledge that death was approaching. In her opinion, a shift in the mindset was needed to avoid hospitalisation in order to have a good farewell. However, if patients and their family did not want to make a shift in their mindset, she accepted that and then treatment in the hospital continued but was appropriate from the perspective of the patient and the family.

GP: “It was impossible in this patient’s case. It simply never switched. It didn’t switch for them, even at the very end.”

Interviewer: “You say that it’s also important for the family to make that switch.”

GP: “Of course you want to grow towards it together, I reckon, towards… So there’s that aim, I think. Growing together towards a fine death where everyone still has the feeling that what had to happen happened. You can say farewell to another. But of course you need to shift into a different mindset for that. Otherwise it’s not possible. And this gentleman was so hyperactive in his delirium as well and mean to his wife so that was a difficult situation at home too. Then you can’t just suddenly say, it’s delirium and that could mean something is up, something that can’t be treated properly, because we don’t know what’s causing this, and now we all have to join in that nice dying process—well of course that won’t work. That switch couldn’t be made.(…) He was admitted to hospital; I believe a lung X-ray was made and they did some blood tests. That showed that it was not at all clear what was causing the delirium, but things were pretty bad. Poor blood labs, very poor kidney function. Very high inflammation rates in the blood. So all in all, very poor physical condition. And when the family heard that, they said well okay, the time has come. He won’t be getting better, and then he was put into a separate room and the family had about one day in which to say farewell and that was really quite good. I don’t think that [the family being able to switch their mindset] would have been possible at home.”

(Case 11, GP, female, age category 40–45)

In addition to the initial discussion with the patient marking that he or she was incurably ill and that life expectancy was limited, additional discussions later on in the illness trajectory could help in switching the mindset to acknowledge that death was approaching and that hospitalisations were mostly not desirable or appropriate. Some GPs and nurses proactively suggested discussing these issues at certain points, such as when there was a functional decline, symptoms accumulated or the patient became more bedbound, because at these points it could be seen that the illness was becoming more severe and death was closer. Some GPs said that for very old patients, it was difficult to hold timely talks about end-of-life issues. On this subject, some ideas arose from the interviews, such as talking about end-of-life issues with all frail patients, all patients aged 80 and older, or when nursing care at home starts. However, for some patients the changes in their condition came unexpectedly fast for the GPs, nurses or family, and then the limited life expectancy and care wishes at the end of life were eventually discussed only shortly before dying. According to GPs and nurses, marking the approach of death and shifting the mindset were important in avoiding hospitalisations at the end of life, however it was difficult to find the right moment to talk about this and achieve acceptance that death was approaching.

#### 2. Being able to provide acute treatment and care at home

In several patient cases, there was an acute symptomatic problem for which the patient was hospitalised. In acute situations, GPs, and family carers too, said that it was important to sit down and take time to discuss all possible options for treatment and care at home or in the hospital. To avoid hospitalisation for the purpose of continuous observation, some nurses said GPs and family carers was not always known that they could respond rapidly and provide night care, especially when the patient was already known by the home care organisation. The comfort of the patient was an essential aspect in acute treatment in the last days of life and palliative sedation could be seen as a good option when there were severe symptoms in the last days of life. One GP, for example, said that he sometimes used intermittent palliative sedation to create a ‘timeout’ to calm everyone down at the patients’ home and think about a solution to the problem.

GP:“Well, then you are the bedrock they can always fall back on, when they get into a panic and think we’re not going to cope. But I explain that they can always call me, day or night. I have the tools, I can apply sedation for instance, simply to create a timeout, to say listen, we’ll calm things down. Let their patients and their family pause for breath so that they can once again accept the unacceptable and go down that road.”(…) “You can give yourself a break and say, well, I’m going to intervene here and then we’ll see later what to do next. Time is a good partner, you know.”

(Case 7, GP, male, age category 55–60)

On the other hand, GPs said that it was often difficult to avoid hospitalisation when the acute situation arose, especially in the out-of-hours service when patients had severe symptoms and GPs had not seen the patient before.

GP:“That’s a bit more difficult when you’re on call, because of course you sometimes get questions like that on call, 2 o’clock on a Saturday night. Well if you, if you have to arrange something then, and often you have the entire family in the room so something has to be done there and then. And then you’re sitting there as a doctor who is a complete stranger with a patient you don’t know at all, whose prior history you don’t know, and then you have to start from scratch at that point; I find that… I don’t enjoy that, I find it very difficult to work like that and there is so much pressure being put upon you and then sometimes you don’t really have any choice other than to get someone admitted to hospital just to be rid of that pressure.”

(Case 16, GP, male, age category 46–50)

Other GPs also mentioned the pressure from the family in stressful situations, such as when patients had acute severe symptoms. Because of the panic and stress in the family, it was sometimes difficult for GPs to resist the pressure from family members who thought that hospital treatment was the best option. In such a situation, taking time and calming everyone down were mentioned as important in avoiding hospitalisation when treatment at home was a good option. So it seemed that knowing the patient and at the moment of an acute situation to take time to discuss the situation and treatment options with patient and family were important in being able to provide acute treatment and care in order to avoid hospitalisations.

#### 3. Anticipatory discussions and interventions to deal with expected severe problems

One of the themes that helped avoid hospitalisations that arose from the interviews was anticipatory discussions about medical decisions at the end of life and interventions to deal with severe problems in the illness trajectory. After it had been marked that death was approaching, and sometimes before that point, some GPs or patients initiated anticipatory discussions about the patient’s preferences regarding staying at home or medical decisions at the end of life. GPs, nurses and family said that patients who were not hospitalised had often expressed the explicit wish to stay at home several weeks before death. GPs and nurses said in the interviews that whether or not to hospitalise was not always discussed directly, but it was touched on indirectly as the preference of patients and their family for staying at home was discussed. In addition to this, GPs and nurses said that anticipatory discussions about medical decisions were often held to clarify the patients’ preferences in the case of resuscitation, euthanasia or palliative sedation. Furthermore, respondents had often anticipated severe problems such as pain or acute bleeding and discussed what patients and their family could do in such a situation, which could avoid acute hospitalisation in such cases.

Nurse: “We get a client at home, often they’ve come from the hospital because that’s where the tests are done. Then we get information transfer from the GPt making clear that this is a terminal client. Well, often we’ll talk then about what we should do if something should happen. What if someone should have a haemorrhage for example or become unwell. Well, then it’s useful to know if we should call the emergency number and then go to the hospital. Or should we consult a out-of-hours general practice, stay at home and do what we can to make things more bearable?”

(Case 13, nurse, female, age category 35–40)

However, GPs and nurses said that symptoms other than pain and bleeding that could become exacerbated or accumulate during the illness trajectory were less often anticipated. Therefore, it was seen that patients were hospitalised for problems other than pain or acute bleeding, such as respiratory problems. However, it was not always possible to anticipate every symptom, according to GPs and nurses.

GP about a male cancer patient (age category 70–75) who had two acute hospitalisations because of a dyspnoea and delirium.

Interviewer: “How could hospitalisation have been avoided?”

GP: “Well, by going through all the possible scenarios, but at the same time that’s not very realistic. At any rate, because there are so many different things that could be done, but some things, well perhaps you could have done them earlier.”

(Case 17, GP, male, age category 40–45)

In addition to anticipatory discussions of the preference for staying at home and what to do in the case of severe symptoms, other interventions were also mentioned in the interviews aimed at preventing hospitalisation after a severe acute situation. Firstly, the GPs sometimes gave their personal phone number. In addition, nurses said that they also gave the number of the nurse in charge if more observation of the patient’s situation was needed. Secondly, registration of the patient’s preferences in the medical chart of the out-of-hours general practice and also documentation of the short life expectancy were considered to be important anticipatory measures for acute situations outside of normal practice hours. Thirdly, if severe symptoms were expected, some GPs put some extra medication in the refrigerator and gave an explanation about how and when to use this. Also, some nurses asked the GP to prescribe ‘as needed’ medication. These anticipatory interventions and the above-mentioned discussions about how to deal with expected severe problems were mentioned as important in helping avoid hospitalisations at the end of life.

#### 4. Guiding and monitoring the patient and family in a holistic way through the illness trajectory

In the interviews, GPs, nurses and family carers talked about the importance of guiding and monitoring because often unexpected problems could arise in the illness trajectory; if these problems were recognised early and there was adequate relief then a hospitalisation could be avoided.

Family carer about her deceased father (not hospitalised) and husband (hospitalised).

“The morphine made my father very calm. It made him sleep an awful lot. But if he got too much morphine, you saw exactly the same as with my husband. Then he would become restless and then he would really get out of control and panic, and it was such a fine balance between those two. That was really my experience with both of them. Because it’s about knowing someone really well and knowing how he responds to medicines, (…) and simply because you (the nurse) are always there and see what’s happening.”

(Case 28, family carer, female, age category 50–55)

For several GPs and nurses, guiding and monitoring meant working in a team that led the patient and the immediate family through the illness trajectory and explained step by step what was happening in the illness trajectory. Some professionals talked about the holistic way in which patients were guided and monitored with regard to multidimensional physical and psychosocial problems that could change slightly in patients and their family, and then hospitalisation could be avoided. From the interviews it emerged that nurses, more than the GPs who were interviewed, were alert to the psychological changes in patients and their family, and worked to strengthen coping mechanisms. Several professionals talked about an intuitive process of guiding and monitoring that needed intensive contact with the patient in order to prevent acute problems.

GP: “If they suddenly get a lot more symptoms all at once, then a lot of people become unsure. So—as a doctor—you try to give these people, for good or for ill, something to hold onto or offer them something, you want to do that to give them some support.”

Interviewer: “What do you want to offer them?”

GP: “Well, that’s a good question. Well, something that gives them the feeling that they may be getting some support or at any rate that they are being taken seriously and that they may be able to have some part in this. To enter that process together, so that they are not left alone, see, and you are trying to do something to guide them through this successfully.”

(Case 27, GP, male, age category 30–35)

Asked in the interviews about the patient’s situation as a whole, sometimes it emerged that professionals did not see the complete picture of their patient. One such example was a patient case where the GP, nurse and family carer were interviewed: it turned out that the nurse, who gave a lot of attention to helping the patient prepare for and accept death, did not give much attention to the partner’s sleeplessness due to the fact that the patient was very restless at night. The reason for hospitalisation in the end was the burden on the partner caused by sleeplessness. In the case of this patient, the GP only had contact with the partner by phone when the partner asked for help, which were often questions about practical aspects, but this GP did not visit the patient and partner to guide and monitor them.

Finally, nurses did not always start the guiding process because some family carers wanted to care for the patient up to the end of life and thought that they did not need the care of a community nurse. Some family carers and also some GPs did not know about the supportive competences of the nurse and that supportive nursing care could start early in the illness trajectory. Nurses said that it was very important for them to start guiding and monitoring patients early in the illness process in order to prevent acute somatic or psychosocial problems that could be a reason for hospitalisation. Some nurses and family carers talked about their concerns when too many nurses cared for a patient and the nurses were not sufficiently qualified: nurses with insufficient training could miss important observations and then fail to guide the patient in an optimum manner. To avoid hospitalisations, GPs and nurses needed to work as a team in guiding and monitoring the patient and family though the illness trajectory from the moment that they acknowledge that death is approaching.

#### 5. Continuity of treatment and care at home

According to some respondents, continuity in treatment and care was considered important in avoiding hospitalisation at the end of life. GPs said that after the medical specialist had marked the approach of death, they wanted to have the lead instead of the medical specialist. Therefore what was needed was one GP and a small number of nurses to provide continuity of treatment and care. Together with the nurses, they wanted to build up a relationship of trust that was a prerequisite for anticipating, guiding and monitoring properly.

GP about a restless man with multimorbidity whose hospitalisation was initiated by a hospital specialist.

GP: “I do feel he was the victim of a lack of continuity care by the GP practice. Otherwise things might have gone differently.”

Interviewer: “Oh? What do you think might have gone differently?”

GP: “You see a difference, then you can take anticipatory measures to deal with what might happen. You’ve built up a relationship and you know what someone’s cognitive status is and how that has changed and then you can take proactive measures, so consult the psychiatrist proactively and get involved, and now I didn’t get involved because I simply didn’t know what his condition was. And that happened in the secondary care without them getting a GP involved.”

(Case 2, GP, female, age category 30–35)

Several patients were treated continuously during the illness trajectory, receiving treatment for pain, nausea or other symptoms or care for a wound or urinary catheter. According to respondents, continuous interaction between the GP, nurse and family carer was needed for this treatment and care in order to prevent acute severe symptoms or other problems that could lead to acute hospitalisations.

As some GPs explained, some patients needed a short hospitalisation for treatment of symptoms, such as for a stent implantation, lung puncture or ascites puncture. If patients needed a short treatment in the hospital it was necessary to stay in dialogue with the medical specialist and discuss when to stop this treatment in the hospital, or go on with this treatment at home in order to prevent long hospitalisations. For other treatments, such as ascites punctures, infusions for antibiotics or blood infusions, it was not always necessary to go to a hospital. Some nurses said that these relatively simple treatments could be provided at home more often if the GP and patient were aware of all the competencies of community nurses. Treatment was often discussed as a technical option that did not result in a heavy physical burden for the patient, but the emotional burden of the treatment in the hospital and the fact that as a result the patient spent less time with the family was less likely to be discussed. One family carer thought that if the emotional burden of the stent implantation had been discussed then his wife would have refused this treatment because she was very anxious about it.

Family carer about the hospitalisation of his wife.

“She was really dreading that, so perhaps she would have said because of that, well I don’t need this anymore. That’s the impression I got.”

(Case 10, family carer, male, age category 80–85)

According to respondents’ accounts of the patients’ situation, continuity of treatment and care often became more intensive when death became imminent. Then, regular observation was required especially if patients needed a syringe pump for medication, because adjustments of medication based on observations might be necessary to treat the symptoms. In addition, nurses explained that nursing care for bathing the patient was often given at the end of the illness trajectory and night care could also be provided, in both cases to relieve the burden of the family carer, which could help the patient to remain at home.

Nurse about a patient who was not hospitalised.

“Then the daughter said, I’m not getting enough sleep at the moment, and that palliative package (extra palliative care from a nurse) had been there for a while by then. Because it had already been applied for by the GP, saying that this man wants to stay at home and eventually he’ll simply need all the care that will let him, in his situation, stay at home and also die at home, if possible.”

(Case 24, nurse, female, age category 40–45)

For GPs, providing continuous treatment of physical or psychosocial problems meant visiting the patient and family regularly, while for nurses it meant providing continuous care and support in order to help the patient stay at home until the end of life.

### Interrelation between the five themes

The interviews revealed not only the five key themes that can be seen as strategies to avoid hospitalisation but also an interrelation between these strategies. The starting point of these strategies was marking the approach of death, which often led to a huge shift in the mindset of patients and their family, and it often took time for a full awareness of this new reality to develop. From that point the benefits and burden of hospitalisations were weighed up in the light of the short life expectancy. Then staying at home, the quality of life and comfort of the patient became more important. After marking the approach of death, the process of anticipating problems, guiding and monitoring in a holistic way and ensuring continuity in treatment and care started, and gradually became more intensive up to the end of life. When interviewees were asked about how these strategies could be optimised to avoid hospitalisation, then it became apparent these key strategies could be improved if implemented in a less fragmented way.

To illustrate the interrelation of the five strategies, three example cases are presented (Cases 1, 2 and 3). In all three cases there was an acute situation, and in two of the cases the patients were hospitalised (Cases 1 and 2). In Cases 1 and 3 we see that it was marked that death was approaching at the point when the medical specialist called the GP to say that there were no effective curative treatment options. This was discussed with the patients but neither patient made a shift in mindset at that moment to acknowledge that death was approaching. In Case 1, the GP anticipated dying but not the illness process as a whole in which dyspnoea in the lung cancer patient could be expected. After that moment the GP was reactive and waited until he was needed. In Case 3 the GP guided and monitored the patient and also offered the continuing support of a nurse; none of this was provided in Case 1. In the embedded continuous care by the GP and nurse in Case 3, it was a small step for the family carer to call the nurse and later on the GP in the event of an emergency.

In Case 2, anticipating acute situations and guiding patient and family though the illness process was difficult because the patient did not want to look at the future, even in his old age. While giving continuous care, the community nurse monitored the patient. However, the nurse did not discuss the patient’s functional decline and refusal to eat and drink with the GP; this could have been another moment for marking the approach of death and a starting point to anticipate the end of life. In Case 2, the family carer thought afterwards that hospitalisation could have been avoided when the acute situation arose if the GP had sat down and discussed all possible options for staying at home.

#### Case 1. Woman with lung cancer, aged 57

The patient is divorced and has two sons aged about 20, one of whom still lives at home. She runs her own business. Three months before the patient’s death, the GP receives a phone call from the specialist saying there are no more curative treatment options. The next day, the GP pays a visit to the patient and at the patient’s request they discuss how the GP can assist her when dying. The GP does not talk about the options for palliative care for the rest of the disease trajectory, nor do they discuss nursing care as the GP judges that the woman and her sons will be able to cope over the next while. The GP tells the woman she can always call him if anything comes up and gives her his mobile number. Afterwards, the GP calls the woman occasionally and asks her if there is anything he can do. The woman wants to continue working for as long as possible, despite her fatigue. The son who lives at home is pleased his mother is taking such a positive view of things and he hopes to keep his mother with him for as long as possible. Three days before she dies, the mother suddenly feels severe tightness in the chest and she calls the medical specialist, with whom she is on good terms. The woman is admitted to hospital for two days, where she receives morphine and oxygen. Then she is sent home with the aim of letting her pass away at home. The next day, she dies in the presence of her two sons.

#### Case 2. Man with dementia, aged 88

The patient lives at home with his younger wife who is still working as a doctor. The patient has been receiving nursing care twice a day for two years. The nursing staff help the man wash, get dressed and take his medication. The patient’s condition is gradually deteriorating; recently he has stopped really wanting to eat or drink anything and he spends most of the day in bed. The GP did make some attempts to discuss the patient’s death with him and his wife but they wanted to leave the future to God. One afternoon, the community nurse finds the patient on the floor and she diagnoses hemiplegia. The GP comes at once and discusses the situation on the phone with the man’s wife. The wife comes straight home from her work and agrees that her husband has hemiplegia; it is difficult to communicate with him and he is confused. The GP has already left but is called on the phone and together they decide to admit the patient to hospital. The woman hopes they may still be able to do something for him in the hospital. Rapid admission is important after a CVA. The patient is admitted to hospital and dies there two days later. When the wife was asked in the interview what she would have done if she had been the GP in charge, she said: “If you want to discuss this difficult problem, you need to allow more time for it. I would talk to the people separately and say that there is a really high risk of this ending in death and the benefit for you if you keep him at home and immediately increase the level of care is that you remain in your own surroundings and he can eventually pass away in his own surroundings. (…) We could do that just as well here at home as we could in the hospital or a hospice or whatever.”

#### Case 3. Patient with prostate cancer, aged 78

The patient is a businessman. He lives with his wife who is in the early stages of dementia and has an adult daughter. The patient is told four months before his death that he has metastatic cancer. Afterwards, he takes part in a medical trial that does not require hospital admission. The daughter experiences it as a big burden because her mother has incipient dementia and does not really understand her husband’s illness. The GP finds it impossible to discuss the approaching death with the patient during the medical trial. The GP does pay regular visits because the informal carers are finding it so tough and he expects the patient to develop severe symptoms. The nursing staff drop in every day because there is a catheter. One month before the patient’s death, the specialist calls the GP to tell him that the medical trial with chemotherapy is being stopped because his blood lab values suggest the cancer is increasing. Then the patient himself takes the initiative to discuss with the GP what they can expect from one another, and they talk about how to achieve a good death. The patient says he may want palliative sedation at the end of his life. The GP and nurse both give their mobile numbers so that they can be called in the event of an emergency. At one point, the patient starts vomiting a great deal. The daughter panics and calls the community nurse, who comes at once. The GP is called as well. Together, they manage to resolve the panic situation. Three days before his death, it seems as if the patient may be getting an intestinal obstruction; he is in a great deal of pain but he does not want to go to hospital. They start administering morphine to stop the pain and later he is sedated. The patient dies at home in the presence of his family.

## Discussion

This qualitative study looked at avoidable hospitalisations from the perspectives of GPs, nurses and family carers. We found an interrelation between five strategies that can help in avoiding hospitalisation. These strategies were: 1) marking the approach of death, and shifting the mindset; 2) being able to provide acute treatment and care at home; 3) anticipatory discussions and interventions to deal with expected severe problems; 4) guiding and monitoring the patient and family in a holistic way through the illness trajectory; 5) continuity of treatment and care at home.

A strength of this study is that in-depth interviews are included from the perspectives of GPs, nurses and family carers. This study provides new insights into the avoidability of hospitalisation at the end of life of patients who reside at home. It has limitations in the generalisability and external validity of the results. However, the findings of qualitative studies are meant rather to help understand processes and practice [20], in this study namely the processes and practice relating to hospitalisation and its avoidance at the end of life. Another limitation of this interview study is the potential recall bias of respondents who were asked about a deceased patient one year to one and a half years after death. To overcome recall bias, GPs and nurses were also asked about other recent patient cases and about what they do in general in such cases, such as how they anticipate hospitalisations in general.

This study presented interrelated strategies for avoiding hospitalisations from the perspective of GPs, nurses and family carers. The starting strategy is that it is necessary to mark the approach of death and to communicate that there are no effective curative treatment options. However, as it is suggested in this study and also shown in other studies, it is often difficult to recognise that death is approaching, especially for non-cancer patients [21]. For professionals, one way of marking the approach of death is is to ask yourself the ‘surprise’ question “Would you be surprised if this patient died in the next year?”, which is part of the internationally renowned Gold Standards Framework (GSF) [22]. The GSF and the Radboud indicators for palliative care needs (RADPAC) also provide clinical indicators for physicians to mark that the patient is in a situation in which the end is approaching [22,23]. In addition to this, this study described that it is important there is a shift in the mindset to acknowledge that death is approaching. However, some patients and family do not to make a shift in the mindset and deny that death is approaching. Research suggests that patients’ denial may have a protective effect on emotional and social outcomes in the quality of life [24]. Therefore, it is recommended that patients are asked whether they prefer to have open communication about the end of life. Irrespective of the patient’s preference for open communication about the end of life, guiding and monitoring by both GPs and nurses are needed in order to be alert to accumulating or acute symptoms in the illness trajectory in the last months of life.

Another strategy in the interrelated strategies that this study has presented is the anticipatory discussions and interventions to avoid hospitalisations. One form of anticipatory discussions is advance care planning, which includes discussions about medical decisions at the end of life. Other studies have indeed shown that holding advance care discussions about medical decisions at the end of life results in fewer hospital deaths [25,26]. Another aspect of this strategy includes the anticipatory interventions. Other studies also show that anticipatory interventions such as the availability of ‘as needed’ medication [27] or information transfer to the out-of-hours GP cooperative [28] can be successful in reducing hospitalisations at the end of life.

The end-of-life care pathway of the Royal College of General Practitioners and the Royal College of Nursing includes several elements of palliative care that are comparable to the strategies as presented in this study, such as identifying that the end of life is approaching, discussions as the end of life approaches, assessment, care planning, review, and coordination of care [29]. In addition to this, our strategies can also be recognised in multidimensional interventions provided by multi-disciplinary teams with highly trained nurses, which have been shown to reduce hospitalisations at the end of life [30,31]. These interventions consisted of timely discussion with the patient and family about their preferences and what might happen at the end of life, and proactive monitoring. These multi-disciplinary teams for palliative care show the importance for GPs and nurses of working in a team. In the Netherlands, new projects have started for palliative care at home (PaTz), in which GPs have rediscovered cooperation with community nurses while before some GPs did not even know about the possibilities of early supportive nursing care [32].

## Conclusions

This descriptive study found five key interrelated strategies for avoiding hospitalisation, namely marking the approach of death and shifting the mindset, being able to provide acute treatment and care at home, anticipatory discussions and interventions to deal with expected severe problems, guiding and monitoring the patient and family in a holistic way through the illness trajectory, and continuity of treatment and care at home. Both GPs and community nurses have their own professional competencies for applying these strategies. Therefore it is recommended that for all patients residing at home, GPs and community nurses should work together as a team from the moment that it is marked that death is approaching up to the end of life. As the five key strategies presented here include several elements of a palliative care approach, we may conclude that palliative care provided by GPs and community nurse can help avoid hospitalisation.
